# What factors matter in the amount of alcohol consumed? An analysis among Brazilian adolescents

**DOI:** 10.1371/journal.pone.0281065

**Published:** 2023-02-21

**Authors:** Lorenzo L. Bianchi, Cristiane da Silva, Lauana Rossetto Lazaretti, Marco Túlio Aniceto França

**Affiliations:** 1 National School of Public Administration, Evidência Express, Brasília, Brazil; 2 Universidade do Vale do Rio dos Sinos, Porto Alegre, Rio Grande do Sul, Brazil; 3 Universidade Federal de Santa Maria, Santa Maria, Rio Grande do Sul, Brazil; 4 Pontifical Catholic University of Rio Grande do Sul, Porto Alegre, Rio Grande do Sul, Brazil; University of Milano–Bicocca: Universita degli Studi di Milano-Bicocca, ITALY

## Abstract

Alcohol consumption in the under-18 age group has been growing in recent years, leading to various health risks. Considering the problems this habit brings, the present study contributes to the literature dedicated to categorizing different types of drinkers. The study objective is to verify the factors associated with the intensity of alcohol use among elementary school students in the year 2015. The dataset came from the National Adolescent School-based Health Survey (PeNSE). The applied methodology was a logit model of sequential response (continuation ratio). The main results are as follows. It was found that being female is associated with a lesser chance of having consumed alcohol in the reference period, however, with a greater chance of consuming five or more doses. Economic condition and formal paid employment are positively associated with alcohol consumption, which increases with the progression of the student’s age. The number of friends who drink alcohol and consumption of products originating from tobacco and illicit drugs, are good predictors of alcohol use by students. The time spent practicing physical activities increased the chance of male students consuming alcohol. The results showed that, in general, the characteristics associated with different alcohol consumption profiles remain similar but differ between genders. Intervention strategies aimed at preventing alcohol consumption by minors are suggested in order to reduce the negative effects of substance use and abuse.

## 1. Introduction

Adolescence is the period of human life from age 10 to 19 where the transition from childhood to adulthood [1] occurs and where alcohol and drug use is known to begin [2]. This period of life is marked by physical, psychological, and cognitive changes and the search for identity and autonomy [3].

Alcohol consumption during adolescence is associated with several harmful health and social consequences. Adolescent drinkers tend to get involved in dangerous situations such as traffic accidents and fights. They are prone to misconduct, hyperactivity, and drug use, and often get into problems with parents, schools, and the police [4, 5]. For girls, alcohol abuse is associated with falling prey to sexual crimes [6]. Alcohol use, for both boys and girls, is associated with risky sexual behaviors, such as having multiple partners and having unprotected sex [7–9]. Another important aspect is the relationship between alcohol consumption and mental health. Some authors, such as Mentzakis et al. [10], bring attention to a large amount of evidence that links alcohol abuse with mental disorders such as depression, anxiety, phobias, and personality disorders. Marschall-Lévesque et al. [11] also indicate the relationship between alcohol consumption and adolescent suicides. Cheng et al. [12] and Weitzman et al. [13] explain that since adolescent drinkers begin drinking early in life, their alcohol consumption period is longer, which leads to increases the chance of developing dependence in the future. In Brazil, alcohol consumption is becoming a major health risk to monitor due to a variety of reasons. Rehm et al. [14] analyzes the 2004 *Global Burden of Disease and Injury* and indicate the existence of an elevated loss of disability-adjusted life-years. According to WHO [15], the male Brazilian population is expected to lose 17.7% disability-adjusted life-years due to alcohol consumption. The World Health Organization 2018 *Global Status Report on Alcohol and Health* projects an 8.3% increase in Brazil’s alcohol consumption per capita until 2025.

Thus, the objective of this study is to verify factors associated with different intensities of alcohol intake by students of the ninth-grade of elementary school during the year 2015, discriminated according to student gender. The econometric model used is a continuation ratio logit.

We chose to estimate a continuation ratio logit based on Fone et al. [46] study of the effects of neighbourhood deprivation and excessive alcohol consumption and binge drinking in Wales (UK). Fone et al. [46] indicate the absence of knowledge in order to distinguish between excess consumption and binge drinking as distinct categories and how those categories may be influenced by neighbourhood deprivation. The authors observe that residents of less deprived neighbourhoods have a greater chance of excessive alcohol consumption. Despite that, Fone et al. [46] also observe that the residents of less deprived neighbourhood also present a lesser risk of binge drinking compared to the observed effect on excessive alcohol consumption. Based on Fone et al. [46] work and their indication of the absence of literature concerned with distinguishing different levels of alcohol intake, this study also aim to better comprehend whether the associations of the studied factors may vary within different population groups of alcohol intake.

The dataset from National Adolescent School-based Health Survey (PeNSE) was used, specifically that of ninth-grade elementary school students of public and private schools. It is noteworthy that in Brazil, selling alcoholic beverages to individuals under 18 years old is forbidden under law 9,294, of July 15, 1996. However, this issue becomes controversial when advertising alcoholic beverages in the media is allowed. Alcoholic beverages are legal drugs, accepted by all social levels, and are easily accessible. [16, 17]. Data from the Brazilian Institute of Geography and Statistics (IBGE) reveal that approximately 34% students enrolled in Brazilian private and public schools had already experimented with alcoholic beverages sometime in their lifetime. In 2015, these proportions increased to approximately 55% of the population in that age range [18]. The highest percentages of alcohol experimentation were observed in Rio Grande do Sul (74.2%), Santa Catarina (73.8%), Paraná (70.2%), followed by São Paulo (69.2%) and Mato Grosso do Sul (68.7%). The lowest percentages were observed in Pará (50.6%), Maranhão (50.8%), and Ceará (53.8%) (IBGE, 2021).

These data about Brazil’s alcohol consumption have important implications for the financing of the national public health system. Coutinho et al. [19] indicate that approximately 8 million dollars (PPP 2010) are spent solely on treating diseases related to alcohol consumption, a sum that is possibly greater considering its role in the spread of other diseases.

This paper contributes to the understanding of alcohol consumption among school adolescents in Brazil. Here, we analyze whether there is a difference in drinking behavior in relation to gender and age groups. Variables such as professional activity, tobacco and drug use, number of friends who drink, duration of physical activity, economic condition, family composition, and parental supervision provide information for the formulation of public health policies for this population. The consumption of alcoholic beverages is a risk factor for the population in general, as it is considered one of the main causes of non-transmissible chronic diseases, accidents, and violence.

The adolescent situation is equally concerning. Although the academic literature presents several studies describing adolescent binge drinking in Brazil (examples include [20–26]), to the best of our knowledge, this is the first work that investigates this subject integrating different types of drinking in a unique empirical framework and using a nationally representative sample. By doing so, the estimated models allow us to analyze different socioeconomic characteristics associated with specific types of drinking and even the different weighting a characteristic may have on distinguishing students who binge drink from moderate drinkers or nondrinkers.

Literature suggests that gender may play an important part in determining how some known characteristics that influence alcohol consumption operate. Nolen-Hoeksema [27] indicates that women drink less alcohol and have fewer alcohol-related problems than men. Hence, women may be less likely to develop alcohol-related problems because they are less likely to have multiple risk factors for these behaviors. Men, unlike women, tend to have characteristics associated with excessive alcohol consumption, including aggressiveness, drinking to reduce distress, lack of behavioral control, sensation-seeking, and antisociality, among others. On the other hand, women are more likely to experience physical harm and sexual assault when drinking alcohol than men, not to mention that heavy alcohol consumption in women is associated with a range of reproductive problems. Moreover, Li et al. [28] and Cohen et al. [29] suggest that females’ own perception of being more closely monitored than males regarding alcohol drinking behavior may contribute to inhibit addictive substance consumption. We address this gap through estimation and comparison of gender specific models in addition to a more general model composed of both males and females students.

Therefore, the analysis presented in this study may contribute to better defining key aspects for formulating public policies aimed at inhibiting alcoholism. Examples include defining the target population among adolescents for preventive measures against alcohol consumption and identifying more effective channels to reach a specific population group that may have a pattern of alcohol consumption that is extremely harmful to health.

Relevant data, such as the evolution of the total number of enrollments in primary schools and the differences observed in the states of the federation can help to better understand the context of Brazilian education. According to the School Census, in 2015, there were 48.8 million enrollments in the 186.4 thousand basic education schools in Brazil, including public and private institutions. The municipal public school’s system holds 46.8% of the basic education enrollments. In the states of Pará, Maranhão, Piauí, Alagoas, and Bahia, the municipal system has a participation in basic education enrollments of more than 60%. In the Southeast region, the greatest participation of municipal system is observed in Espírito Santo (55.8%) and the smallest in São Paulo (37.8%). Although the state system holds 16.5% of the schools, its participation in enrollments in basic education is 33.9%. In the Midwest region, considering the number of enrollments, the state system exceeds the other systems, with a participation of 44%. The private system corresponds to 18.6% in the total number of enrollments in basic education, while the federal system corresponds to less than 1%. Even though 34.7% of Brazilian schools are located in rural areas, they hold only 11.7% of the enrollments (5.7 million). It is noteworthy that the differences in urbanization between the regions of the country are reflected in the participation of the rural zone in enrollments. For example, while in Maranhão 35% of enrollments occur in rural areas, in São Paulo the percentage is less than 2% [30].

There were 12.4 million enrollments in the final years of elementary school, 14.5% of which were in private schools. The private system grew 14% in seven years. The state education system had 5.4 million enrollments, with a participation of 43.6% in enrollments, dividing the responsibility of providing public education with the municipalities, which had 5.2 million enrollments in the final years of elementary school (41.7%) in 2015. The states of São Paulo and Mato Grosso have the lowest rates of age/grade distortion, with 89% and 86% of the municipalities with rates lower than 20%, respectively [30].

This study is organized in six sections following this introduction. In section two, the literature review is presented. In three, the data and methodology are exposed. Sections four and five of this paper report and discuss the estimations results. The last section presents a brief overview and suggestions for future research.

## 2. Literature review

Given the relevance of studying alcohol consumption among adolescents, this section discusses the main factors that lead to alcohol consumption. Among Spanish adolescents, alcohol consumption is mainly due to the influence of friends, smoking, positive expectations from the use, and being male [31]. In assessing the effects of alcohol consumption on popularity and improved social interaction in the United States, Ali et al. [32] found that those who drink are more likely to be having friends and increments in their friend network. For Kumar et al. [33], higher income level and living in urban areas were positively associated with increased alcohol consumption.

However, these characteristics may influence alcohol consumption differently depending on the intensity of the consumption [31]. The predictive variables of individuals who consume alcohol sporadically differ from those who consume it frequently and have previous problems and risks with alcohol consumption.

Rehm et al. [34] point out to the increasing scientific interest in reporting and analyzing different drinking patterns such as volume, frequency, and quantity of alcohol consumption. Berggren and Sutton [35] suggest that there is a clear rationale for disaggregating these behaviors instead of analyzing total volume of alcohol intake as there exists considerable evidence relating different alcohol consumption patterns to specific problematic outcomes. Rehm et al. [36] present a systematic literature review on patterns of drinking and the burden of disease, for example, and indicate the existence of a clear linkage between the occurrence of more damaging consequences of alcohol consumption, from injuries and accidents to heavy drinking occasions with intoxication.

One important drinking pattern that specially affects adolescent health is binge drinking (i.e., the consumption of five or more alcoholic drinks for males and four or more alcoholic drinks for females on the same occasion). Courtney and Polich [37] highlight the lack of definitional precision regarding the term “binge drinking”. In this work, we adopt the US National Institute on Alcohol Abuse and Alcoholism’s definition of binge drinking as the consumption of five or more alcoholic drinks for males and four or more alcoholic drinks for females on the same occasion on at least one day in the past month [38]. Binge drinking is a specially concerning health risk factor for adolescent health for several reasons. Miller et al. [5] highlight that alcohol consumption is a major risk factor associated with leading causes of death among young people, such as unintentional injury, suicide, and homicide. Hingson and Zha [39] indicate that many adolescent health risk behaviors (like illicit drug use, tobacco-based product consumption, and violent behavior) are more frequently observed in adolescents who binge drink. Chung et al. [40] and Kuntsche and Gmel [41] indicate that greater chances of binge drinking are usually observed in this period of life. Moreover, La Fauci et al. [42] indicate that binge drinking among adolescents has gone through a sharp upsurge in recent times.

Given the importance of the subject, some studies have dedicated themselves to analyzing the characteristics that distinguish binge drinkers from other types of drinkers. Examples include Hill et al. [43] and Tucker et al. [44], who investigate the developmental course of alcohol use from adolescence to early adulthood; Lac and Donaldson [45], who carry out a discriminant analysis to categorize drinker types between non-drinkers, moderate, and binge drinkers; and Fone et al. [46], who analyze the socioeconomic patterning of excess alcohol consumption and binge drinking in the UK.

Commenting on studies that used discriminant analysis, Lac and Donaldson [45] indicate that previous efforts to categorize drinker types have been limited in several ways. These limitations include the exclusion of nondrinkers as an investigated category and the absence of models that evaluate in a unique way the different responses between males and females.

Based on the work of Harris and Zhao [47] about zero-inflated outcomes on ordered models, the exclusion of nondrinkers in an empirical framework implies the exclusion of two distinct types of drinkers. The first type includes those individuals who abstain from alcohol consumption due to a disinterest in alcohol. The second type includes individuals who want alcoholic beverages but did not consume those goods during the study period for external reasons, like elevated prices or difficult access. Excluding these two groups of drinkers from empirical analyses may potentially bias the estimation results due to missing data in the selection process. We address this limitation through the analysis of an ordered scale of drinking patterns composed of four different alcoholic consumption patterns: students who never consumed alcohol in their lifetimes, who have not consumed alcohol for a brief period of time, who consumed a moderate amount of alcohol, and binge drinkers.

## 3. Methods

### 3.1 Data and participants

We use data from the 2015 Survey of National Adolescent School Health (PeNSE). PeNSE 2015 is the third edition of a nationally representative survey of students enrolled in the ninth grade in Brazilian public and private elementary schools. The survey contains questions along themes related to adolescent health risk factors, namely the consumption of addictive substances, nutrition and dietary habits, practice of physical activities, and others. Brazil’s Ministry of Health commissions and funds the Brazilian Institute of Geography and Statistics (IBGE) to design the survey and perform data collection. PeNSE’s sampling units are selected from the school’s sampling structure as found in Ministry of Education’s School Census.

PeNSE 2015 comprised of two independent probabilistic samples. The first sample is composed of students enrolled in the ninth grade (the ideal age to achieve this education level is 14 years, although in Brazil, age/grade distortion is observed in 16% of ninth-grade students) of private and public primary schools with at least fifteen students registered in the ninth grade. The second sample target population were students enrolled in the sixth to ninth grades of primary schools and students enrolled in any level (first, second, or third years) of secondary schools with at least fifteen students enrolled in these grades. Despite having a greater coverage of educational levels, the second sample does not accurately identify student population heterogeneity along different state levels and was collected with the major intent of comparison with indicators of the World Health Organization’s Global school-based student health survey (GSHS). With these factors in mind, only data from the first sample of the 2015 PeNSE was analyzed.

Survey design consisted of a multistage sampling plan. The Brazilian national territory was divided into 53 geographic strata. Using data from the 2013 School Census (the most recent survey on number of schools considered during sample planning), the participant schools were distributed among these 53 strata according to three factors: geographic location, whether they are public or private schools, and the existing number of ninth grade classes. The allocation was implemented in this fashion to guarantee that the presence of both public and private schools in the sample was proportional to their presence in the sampling frame. In the selected schools, the ninth-grade classes were randomly selected, and all students were invited to participate in the study.

We planned to conduct interviews in 3,160 schools. However, there was a sample loss of 3.8% as 120 schools did not participate. Thus, in total, the sample analyzed includes 3,040 schools. After randomizing the secondary sampling units (random selection of one class for schools that reported having two ninth grade classes and random selection of two classes in case of three or more ninth grade classes), 102,301 questionnaires were initially administered, which represents the ninth-grade population with an approximate maximum error of 3% and a 95% confidence level. Although the target population consists of ninth graders, 1,540 students informed that they were enrolled in grades other than the ninth grade. These cases were excluded from the analysis carried out in this study. Data collection was carried out through a Personal Digital Assistant without the interference of an interviewer. The choice of using ninth grade students as the target population of the research is justified by the existence of a minimum level of literacy that allows them to comprehend the self-applied questionnaire. Survey questionnaire and data collection are approved by the National Commission of Ethics in Research (Conep), the governmental institution responsible for normative regulation of health research involving human beings. At the beginning of the 2015 PeNSE, participants were asked to declare their consent to participate in the research. The 2015 PeNSE non-identifiable dataset is available to the public and can be downloaded at IBGE’s web page [48].

### 3.2 Alcohol outcome measure

Primarily, PeNSE 2015 participants were questioned on whether they had ever consumed alcoholic beverages in their lifetime. Students who gave an affirmative response to this question were additionally asked if they had drunk alcoholic beverages in the previous thirty days and how many drinks they consumed on these occasions. Based on these responses and their sex, PeNSE 2015 students were assigned into four groups of alcohol consumption outcome: never-drinkers, non-drinkers, moderate drinkers, and binge drinkers.

Both male and female students who declared that they had never consumed alcoholic beverages during their lifetime were classified as never-drinkers. Male or female students who had not consumed alcohol in the previous thirty days before filling up the questionnaire (but consumed alcohol during their lifetime) were placed in the non-drinker category. Male students who declared drinking less than five doses of alcohol were allocated to the moderate drinker category. Female students who declared to have drunk less than four doses of alcohol were also allocated to the moderate drinker category. Both male and female students who responded that they had drunk above the respective thresholds (five or more drinks for males and four or more drinks for females) were assigned as binge drinkers. This classification of binge drinking was adopted following the US National Institute on Alcohol Abuse and Alcoholism [38].

### 3.3 Independent variables

Models were estimated using independent variables described in S1 Table. These variables were chosen through an analysis of relevant literature. Students’ basic characteristics, such as age, sex, and ethnicity, were included to capture possible demographic trends related to alcohol consumption initiation and binge drinking. The authors choose to use age groups rather than a continuous age variable because a student’s age information in PeNSE 2015 is truncated at eleven and nineteen years old. For more details, see Pechansky et al. [49]. Likewise, we controlled for the possible influence of extracurricular activities, like a paid or unpaid occupation (such as internships, part-time jobs, etc.) and the number of hours spent on physical activities not related to physical education (PE) class. The estimated models controlled for the consumption of tobacco-based products or illicit drugs in the previous thirty days and also for the student’s perception of how many of his or her friends consumed alcoholic beverages.

The estimated models also controlled for some household and school characteristics as Brazil is a country with high social inequality (the 2015 GINI index is 0.491, according to the Brazilian Institute of Geography and Statistics–IBGE [50]. Owing to inequality, students were exposed to different environments that offered varying levels of favorability for the consumption of alcoholic beverages. Factors related to social environment included whether the student studied in a public or full-time school, the school’s geographic location, living with one or both parents or guardians, and the number of people with whom the student shared his/her residence.

Three variables listed in S1 Table were constructed through principal component analysis (PCA): household economic status, parental supervision, and mental health condition. These analyses were made with the aim of capturing some otherwise non-observable information and summarizing the influence of a group of characteristics related to the described dimensions of PeNSE 2015 in the student’s life.

The household economic status variable was based on whether the student’s household had a landline telephone, a computer, internet access, car, motorcycle, a housekeeper, and on whether the student had a personal mobile phone exclusively for his or her own use. The parental supervision variable was based on how often (never, rarely, sometimes, most of the time, always) the student’s parents knew what the student was doing during free time, checked their homework, and went through their belongings. The mental health condition was measured based on how often (never, rarely, sometimes, most of the time, always) the student felt upset, bothered, hurt, offended, or humiliated by his or her classmates, felt lonely, and could not sleep at night because something was bothering him or her. The generated variables have a distribution with a mean of zero and a standard deviation of two. Which generates an indicator with positive and negative numbers. A higher value on these variables indicates a student having better household economic status, more protective parents, and a worsened mental health condition. Only the principal component of each these PCAs was used to resume the respective dimension and the results of these analyses are presented in the S2 Table.

It is important to point out that the variables listed in S1 Table are based on students’ answers to the 2015 PeNSE questionnaire. Consequently, it is not possible to ignore the presence of tentative answers due to intentional omission of information or failure to accurately record some events.

### 3.4 Methodology

We used a partially constrained continuation ratio logit model [51] to analyze the socioeconomic determinants of students’ alcohol consumption. It is also known as sequential response model [52].

In this paper, we have chosen the continuation ratio forward model which compares a certain category with higher categories, and thus estimates the chances of a student being in a certain category versus being above that category. For example, the ordinal response variable (never drank in life, no alcohol consumption in the reference period, moderate drinkers, binge drinkers) has four categories, the continuation ratio forward model compares category 1 with categories 2, 3, and 4; category 2 with categories 3 and 4; and category 3 with category 4 [53]. In this sense, a subject must pass through each antecedent category before reaching a latter outcome. Examples include level of educational attainment [54] and self-rated health [55]. Continuation ratio model was already previously used to study alcohol consumption by Fone et al. [46] who analyzed associations between level of neighborhood deprivation and daily alcohol consumption across the UK Department of Health guideline categories of alcohol consumption. Considering that the forward continuation ratio model estimates the chances of a student being in a certain category *j* versus being above that category, here is how it can be expressed:

$$ln(\frac{P(Y=j|x_{1},x_{2},\ldots ,x_{p})}{P(Y>j|x_{1},x_{2},\ldots ,x_{p})})=\alpha_{j}+(-\beta_{1}X_{1}-\beta_{2}X_{2}-\ldots -\beta_{p}X_{p})$$

where $P(Y=j|x_{1}{,x}_{2},\ldots ,x_{p})$ is the conditional probability of being in the category *j* (never drank in my life, no alcohol consumption in the reference period, moderate drinkers, binge drinkers) conditional on being in or above that category given a set of predictors, i.e., $P(Y=j|Y\geq j);j=1,2,\ldots J-1;\alpha_{j}$ are the cut-off points, and *β*_1_,*β*_2_,…,*β_p_* are the logit coefficients. The continuation ratio model assumes that the logit coefficients for each predictor are the same across all ordinal categories, so this model is also called the restricted continuation ratio model [53].

In this study, we choose to adopt a scale of increasingly undesirable outcomes. In doing so, we consider the absence of alcohol consumption over a student’s lifetime as the lowest (most preferable) outcome of this scale, followed by not consuming alcohol over the reference period as the second most desirable outcome, and the group of moderate drinkers as the third most desirable option. At the end of the scale lies binge drinking, which assumes the position of least preferable outcome of our ordered scale. Three models were estimated: one for each sex (male and female) and a complete model composed of both boys and girls using sex as a covariate. All estimates were made considering the sample weight assigned to each student as a result of the stratified complex sampling process of the research.

Following Cole and Ananth [51], we test for the existence of proportionality of odds. This hypothesis refers to the existence of equivalent (proportional) effect of a known covariate in the transition between different categories that makes up an ordered scale. Under this hypothesis, a covariate is expected to present the same statistical effect on each transition of state (for example, from never-drinkers to non-drinkers, moderate drinkers, and binge drinkers, and so on) in the estimated models. We use Brant [56] test to identify the presence of proportionality of odds. A 5% significance on the test statistic was used to determine the presence of symmetric effects for each of the variables described in Table 1.

**Table 1:** Description of the sample by alcohol consumption categories.

	Never-Drinkers	Non-drinkers	Moderate Drinkers	Binge Drinkers	Total
Sex					
Boys	46.6%	30.5%	18.1%	4.8%	48,284
Girls	45.4%	28.6%	19.7%	6.3%	51,941
Household economic status					
Mean	-0.053	0.011	0.045	0.232	
Age groups					
13 years old or less	57.9%	26.3%	13.4%	2.4%	16,917
Between 14 and 17 years old	43.8%	30.1%	20.0%	6.2%	82,018
18 years old or more	30.9%	32.5%	27.1%	9.5%	1,290
Racial group					
Caucasian	46.6%	28.8%	19.1%	5.5%	33,260
Black	43.1%	30.1%	20.3%	6.6%	12,532
Asian	44.3%	31.3%	18.3%	6.1%	4,495
Multiracial	46.5%	29.7%	18.5%	5.3%	46,111
Indigenous	45.4%	30.1%	18.8%	5.7%	3,725
Extracurricular activities					
Only studies	48.2%	29.2%	17.7%	4.8%	87,645
Studies and unpaid occupation	36.2%	32.6%	24.3%	6.8%	1,054
Studies and paid occupation	29.7%	31.3%	27.8%	11.1%	11,476
Time spent doing physical activities					
Mean	1.870	2.018	2.096	2.341	
Tobacco-based products consumption					
No	49.4%	30.5%	16.5%	3.5%	92,329
Yes	5.3%	18.2%	47.2%	29.3%	7,868
Illicit drug use					
No	47.7%	30.1%	17.9%	4.3%	96,278
Yes	2.8%	14.6%	45.9%	36.7%	3,812
Number of friends who drink alcoholic beverages					
None	76.4%	19.0%	4.2%	0.4%	20,181
A few	47.9%	33.8%	16.4%	1.9%	27,790
Some	35.4%	35.8%	24.1%	4.8%	23,718
Most	19%	29%	35%	17%	17,031
All	11.6%	21.0%	37.4%	30.0%	2,737
Mental health condition					
Mean	-0.247	0.109	0.283	0.497	
School management					
Private School	49.3%	28.5%	16.9%	5.3%	20,752
Public School	45.1%	29.8%	19.5%	5.6%	79,473
Full time school					
No	45.8%	29.8%	18.7%	5.5%	77,549
Yes	46.0%	28.4%	19.8%	5.6%	22,221
Household family structure					
Does not live in the same household	35.2%	33.9%	22.4%	8.5%	6,471
Lives with one parent (mother or father)	39.9%	32.3%	20.9%	6.9%	35,967
Lives with both parents	51.0%	27.3%	17.3%	4.4%	57,674
Parental supervision					
Mean	0.246	-0.148	-0.238	-0.438	
Number of people living in the same household					
Mean	4.588	4.497	4.513	4.413	
Geographic region					
North	48.6%	30.4%	16.6%	4.5%	23,004
Northeast	49.9%	27.8%	17.3%	5.0%	35,957
Southeast	44.7%	29.3%	19.7%	6.3%	17,501
South	34.7%	31.0%	26.6%	7.6%	9,747
Midwest	41.1%	31.6%	20.7%	6.6%	14,016
Total	46,079	29,588	18,977	5,582	100,225
	45.9%	29.5%	18.9%	5.5%	

Source: Prepared by the authors based on information from PeNSE 2015.

The results of the estimated Brant [56] tests are presented in the (S3 Table) and suggests that, in general model, a students sex (being a female), being between 14 and 17 years old, being asian or multiracial, emotional state, attending to a public school, living with both parents, number of people who live in the same household, time spent on physical activity, tobacco and illicit drugs consumption and all variables describing the number of friends who drink alcoholic beverages and geographic location have violated the proportionality of chances assumption. An equality restriction was applied to the maximum likelihood process for the remaining variables who did not violated the null hypothesis of proportionality of odds in the estimated model. The same process of diagnostic and estimation was applied to gender segmented models.

## 4. Results

### 4.1 Descriptive analysis

Table 1 summarizes sample characteristics over alcohol consumption categories. The data described represent the percentage distribution of students in each category, the exact number of the distribution is presented in the S4 Table. Alcohol consumption categories indicate that almost half of the analyzed sample is composed of students who declared never having consumed alcoholic beverages over their lifetime. The least populated category was of binge drinker students who made up 5.5% of the described sample. Overall, the sample analyzed is composed mainly of multiracial and multiethnic students (with Caucasian students being the second largest category); female students; students in the age range of 14–17 years; and students enrolled in public schools whose main occupation was studying.

Table 1 suggests a clear developmental pattern concerning some of the previously described covariates. Better household economic conditions were associated with higher levels of alcohol consumption. Worse levels of mental health were associated with higher alcohol consumption (variable was constructed through principal component analysis (PCA), a higher value on these variable indicates a student having a worsened mental health condition). Participants who declared consuming illicit drugs and tobacco-based products in the previous thirty days were more likely to declare a higher consumption of alcohol and to be allocated to the moderate and binge drinker categories. A similar pattern is observed in the relationship between the number of peers who consumed alcohol. Table 1 indicates that having more friends who drink alcoholic beverages is more strongly associated with being a moderate or binge drinker.

Regarding parents’ or guardians’ influence on students’ alcohol consumption, it is shown that living in the same household with both parents was more frequently associated with a student never ever having consumed alcohol. The index of parental control also points to the same conclusion as descriptive statistics indicates a decreasing association between higher levels of parental supervision and the likelihood of a student being assigned to higher ordered categories of alcohol consumption.

### 4.2 Continuation ratio models

Tables 2 and 3 present the results of the estimated continuation ratio models. Results of both tables are displayed in odds ratios so that coefficients above unity indicate a higher chance of progressing to higher levels of alcohol consumption and coefficients below unity indicate a decreasing chance of moving on to higher categories (higher chance of a participant being in the current category). Grouped results indicate variables that presented proportionality of chances according to Brant [56] test. These are the cases of students having a professional occupation besides studying and living with a single parent (Table 2) and studying in a public school or performing unpaid activity for both models of Table 3.

**Table 2:** Results of the sequential logit model (in odds ratios).

	General		
	(1)	(2)	(3)
Independent variables			
Student’s Sex (1 = woman)	0.945*	1.264***	1.423***
	(0.0283)	(0.0481)	(0.0918)
Economic condition of household	1.090***	1.057***	1.124***
	(0.0124)	(0.0150)	(0.0276)
Student’s age			
Between 14 and 17 years	1.238***	1.192***	1.612***
	(0.0481)	(0.0640)	(0.166)
18 years or older	1.749***		
	(0.138)		
Racial group			
Black	1.027		
	(0.0348)		
Asian	0.959	0.981	1.017
	(0.0620)	(0.0831)	(0.153)
Multiracial	1.007	1.016	0.922
	(0.0319)	(0.0397)	(0.0590)
Indigenous	0.988		
	(0.0523)		
Extracurricular activities			
Studies and takes up UNPAID occupation	1.271***		
	(0.107)		
Studies and takes up paid occupation	1.450***		
	(0.0427)		
Physical activity time	1.022***	1.017**	1.041***
	(0.00600)	(0.00748)	(0.0120)
Tobacco consumption	7.715***	4.103***	1.991***
	(0.781)	(0.271)	(0.142)
Illicit drugs consumption	4.976***	2.358***	1.564***
	(1.068)	(0.267)	(0.141)
Number of friends who drink alcoholic beverages			
Few	3.211***	2.221***	0.787
	(0.128)	(0.169)	(0.163)
Some	4.569***	2.884***	1.348
	(0.191)	(0.217)	(0.267)
Most	8.781***	5.580***	3.071***
	(0.430)	(0.427)	(0.598)
All	13.65***	7.125***	4.446***
	(1.584)	(0.824)	(0.929)
Student’s emotional state	1.206***	1.051***	1.045*
	(0.0147)	(0.0153)	(0.0249)
Public school student	1.280***	1.006	1.178*
	(0.0520)	(0.0516)	(0.100)
Full time student	0.997*		
	(0.00139)		
Household family structure			
Lives with one parent (father or mother)	0.912**		
	(0.0392)		
Lives with both parents	0.672***	0.880**	0.728***
	(0.0312)	(0.0447)	(0.0519)
Level of supervision of parents or guardians	0.744***	0.980	0.905***
	(0.00955)	(0.0155)	(0.0235)
Number of people in the household	0.985	1.020**	0.979
	(0.00920)	(0.00940)	(0.0150)
Geographic region			
Northeast	1.033	1.260***	1.043
	(0.0330)	(0.0526)	(0.0783)
Southeast	1.160***	1.222***	0.882
	(0.0463)	(0.0618)	(0.0775)
South	1.701***	1.528***	0.817**
	(0.0791)	(0.0838)	(0.0756)
Midwest	1.260***	1.056	0.907
	(0.0470)	(0.0493)	(0.0738)
Cutoffs	0.273***	0.132***	0.0569***
	(0.0232)	(0.0155)	(0.0150)
Observations	89,851		

Note: 1. Standard error in parentheses. 2.

*** p <0.01

** p <0.05

* p <0.1. (-) indicates variables not included in the model. 3. The first column of each model demonstrates the chance of a transition between never-drinkers to non-drinkers, moderate, or binge drinkers; the second column the shift from nondrinkers to moderate or binge drinkers; and the third column the conversion from moderate to binge drinkers. 4. The sample is composed of students enrolled in the ninth grade. 5. Results of both tables are displayed in odds ratios so that coefficients above unity indicate a higher chance of progressing to higher levels of alcohol consumption and coefficients below unity indicate a decreasing chance of moving on to higher categories (higher chance of a participant being in the current category). Grouped results indicate variables that presented proportionality of chances according to Brant [56] test.

Source: Prepared by the authors

**Table 3:** Results of the sequential logit model (in odds ratios).

Variables	Female (girls)			Male (boys)		
	(1)	(2)	(3)	(1)	(2)	(3)
Economic condition of household	1.079***	1.072***	1.101***	1.091***	1.048**	1.172***
	(0.0160)	(0.0200)	(0.0326)	(0.0171)	(0.0204)	(0.0417)
Student’s age						
Between 14 and 17 years	1.219***	1.237***	1.601***	1.267***	1.090	1.665***
	(0.0635)	(0.0854)	(0.214)	(0.0752)	(0.0955)	(0.322)
18 years or older	1.949***	1.213	2.185***	1.874***		
	(0.372)	(0.222)	(0.619)	(0.206)		
Ethnicity						
Black	1.118**			1.011	0.822**	1.216
	(0.0533)			(0.0643)	(0.0662)	(0.168)
Asian	1.060			0.872*		
	(0.0684)			(0.0699)		
Multiracial	1.046			0.981	0.901*	1.025
	(0.0355)			(0.0454)	(0.0538)	(0.106)
Indigenous	1.030			0.948		
	(0.0727)			(0.0748)		
Extracurricular activities						
Studies and unpaid occupation	1.356**			1.232**		
	(0.196)			(0.122)		
Studies and paid occupation	1.575***	1.211**	1.315**	1.507***		
	(0.114)	(0.0947)	(0.145)	(0.0576)		
Physical activity time	1.009	1.009	1.017	1.026***	1.023**	1.061***
	(0.00950)	(0.0114)	(0.0175)	(0.00759)	(0.00984)	(0.0167)
Tobacco-based product consumption	10.24***	4.536***	1.961***	6.503***	3.777***	2.005***
	(1.708)	(0.434)	(0.189)	(0.832)	(0.344)	(0.213)
Illicit drug consumption	3.623***	2.606***	1.537***	5.935***	2.262***	1.602***
	(1.209)	(0.461)	(0.195)	(1.569)	(0.335)	(0.208)
Number of friends who drink alcoholic beverages						
Few	3.484***	2.211***	0.956	3.000***	2.198***	0.697
	(0.205)	(0.244)	(0.302)	(0.164)	(0.229)	(0.196)
Some	5.113***	2.743***	1.709*	4.101***	3.040***	1.137
	(0.307)	(0.296)	(0.521)	(0.239)	(0.316)	(0.299)
Most	10.20***	5.508***	3.789***	7.385***	5.581***	2.660***
	(0.687)	(0.599)	(1.140)	(0.535)	(0.599)	(0.687)
All	18.96***	6.552***	5.897***	9.793***	7.701***	3.397***
	(2.915)	(1.003)	(1.876)	(1.672)	(1.364)	(0.952)
Student’s emotional state	1.244***	1.053***	1.088***	1.159***	1.045*	0.991
	(0.0202)	(0.0197)	(0.0333)	(0.0212)	(0.0239)	(0.0391)
Public school student	1.179***			1.153***		
	(0.0471)			(0.0513)		
Full time student	1.001	0.987***	1.006	0.998		
	(0.00280)	(0.00374)	(0.00782)	(0.00221)		
Household family structure						
Lives with one parent (father or mother)	0.930			0.880**		
	(0.0558)			(0.0529)		
Lives with both parents	0.673***	0.942	0.766***	0.659***	0.825***	0.669***
	(0.0440)	(0.0655)	(0.0729)	(0.0423)	(0.0599)	(0.0713)
Level of supervision of parents or guardians	0.730***	0.966	0.903***	0.763***	0.988	0.912**
	(0.0132)	(0.0209)	(0.0307)	(0.0138)	(0.0229)	(0.0369)
Number of people in the household	0.999			0.995		
	(0.00936)			(0.00941)		
Geographic region						
Northeast	0.999	1.379***	1.069	0.971	1.243***	1.339***
	(0.0411)	(0.0689)	(0.0873)	(0.0409)	(0.0675)	(0.130)
Southeast	1.115**	1.322***	1.104	1.107*	1.180**	0.880
	(0.0591)	(0.0822)	(0.106)	(0.0583)	(0.0782)	(0.0998)
South	1.830***	1.678***	0.911	1.498***	1.387***	1.041
	(0.114)	(0.114)	(0.0907)	(0.0925)	(0.0992)	(0.124)
Midwest	1.195***			1.084**		
	(0.0457)			(0.0431)		
Cutoffs	0.233***	0.141***	0.0468***	0.325***	0.162***	0.0498***
	(0.0244)	(0.0206)	(0.0165)	(0.0353)	(0.0251)	(0.0172)
Observations	46,748			43,103		

Note: 1. Standard error in parentheses. 2.

*** p <0.01

** p <0.05

* p <0.1. (-) indicates variables not included in the model. 3. The first column of each model demonstrates the chance of a transition between never-drinkers to non-drinkers, moderate, or binge drinkers; the second column the shift from nondrinkers to moderate or binge drinkers; and the third column the conversion from moderate to binge drinkers. 4. The sample is composed of students enrolled in the ninth grade. 5. Results of both tables are displayed in odds ratios so that coefficients above unity indicate a higher chance of progressing to higher levels of alcohol consumption and coefficients below unity indicate a decreasing chance of moving on to higher categories (higher chance of a participant being in the current category). Grouped results indicate variables that presented proportionality of chances according to Brant [56] test.

Source: Prepared by the authors

Table 2 displays the continuation ratio model estimation results of a combined sample of male and female students. Regarding demographic trends, results indicate that female students were less likely to begin drinking. In cases where the drinking had already begun, female students also presented an elevated chance to declare they had consumed alcohol in the reference period. It should be noted that these chances were increasing in a sense that girls were more likely to binge drink than boys.

Older students presented an elevated chance of consuming alcoholic beverages. The risk of consuming alcoholic beverages varied in students aged fourteen to seventeen across different categories and it was constant in students aged eighteen and over. The consistency of the odds ratio observed in the last age group may relate to the fact that 18 years is the threshold legal age for a person to be allowed to buy alcoholic beverages in Brazil.

Students who were engaged in a professional activity displayed a higher chance of beginning to drink and consuming more alcohol. It should be noted that being engaged in a paid occupation indicated a higher chance of consuming alcohol than not being paid to work.

The consumption of tobacco-based products and illicit drugs and the number of friends who also consumed alcohol were important predictors of student’s alcohol consumption. Table 2 indicates that consumption of tobacco-based products and illicit drugs presented a decreasing positive effect. This result suggests that consumption of drugs is more common when the initiation into alcohol consumption has occurred since greater effect is observed in the first stage.

A similar pattern was observed regarding the number of friends who also consumed alcohol. Results suggest that an increasing number of friends who also consume alcohol is associated with an increasing chance of a student consuming alcoholic beverages. It is worth noting that students who had a few or some friends who consumed alcohol were not vulnerable to becoming binge drinkers, although it increased the chance of being initiated into alcohol and its consumption over the reference period. In that regard, all or most friends being alcohol drinkers was associated with higher chance of a student being a binge drinker.

The results of estimations divided by sex (male and female) are presented in Table 3. These models suggest the existence of some differences related to the effect a covariate may play on a student’s alcohol consumption. A more visible example of these differences is the contrast between the amount of time boys and girls spend on physical activities and between living with just one parent.

The results in Table 3 indicate that amount of time spent on physical activities increased male students’ chance of consuming alcohol. The presented results also indicate that time spent on physical activities did not influence female student’s alcohol consumption. A similar pattern of results was observed for students living with only one parent in the sense that it decreased male students’ probability of consuming alcohol and did not influence female students’ chance of consuming alcohol.

Apart from Indigenous male students, non-Caucasian ethnicities were associated with a decreased chance of consuming alcohol in the reference period. Other than that, being a non-Caucasian male student did not influence other aspects of alcohol consumption behavior, such as the probability of initiation or binge drinking. Black female students presented a positive chance of progressing along the alcohol outcome measure. This result indicates that black female students exhibit a greater chance of initiating alcohol consumption, consuming alcohol during the reference period, and being binge drinkers altogether.

Strong predictors of alcohol consumption include number of friends who consume alcohol and consumption of tobacco-based products and illicit drugs, and their importance in determining alcohol consumption persisted in both male and female individually estimated models.

## 5. Discussion

The present study found that different levels of consumption are associated with different characteristics in Brazilian students in terms of magnitude and direction, which may vary between boys and girls. As this association had not been studied in Brazil, this is an important finding since it reveals that implementing strategies focused on preventing a particular behavior, like adolescent casual day-to-day drinking, may have a positive side effect on other related habits related to alcohol consumption such as adolescent binge drinking or alcohol initiation. Following on that, the very knowledge that the characteristics associated with these different alcohol consumption profiles do not differ may be a gain by itself. Knowing that these characteristics remain the same could possibly simplify the process of public policy formulation for alcohol consumption prevention agenda considering that implementing an action focusing on a certain alcohol consumption profile will not present negative side effects for others.

Specifically, based on the sole analysis of the methodology used to analyze the alcohol consumption profiles studied in this paper, one could expect the existence of three patterns or groups of results concerning the influence of the studied characteristics on the classification of the analyzed students in the groups of never-drinkers, non-drinkers, moderate drinkers, and binge drinkers.

The first group corresponds to some characteristic having a tenacious effect on possibly influencing students to present an unhealthier pattern alcohol consumption. Variables that exemplify this logic are household economic status and the consumption of tobacco or illicit drugs, which remained significant across all estimations and only presented a positive effect in the sense of elevating the chances of a student presenting more unhealthy level of alcohol consumption. The second group is represented by the case of characteristics of parental supervision. The model that combines both male and female students indicated that more attentive parents reduced the chances of a student being a binge drinker and experimenting with alcohol but did not interfere with the chances of the transition from nondrinker to moderate or binge drinker. The third pattern of results would be the case of a variable having a conflicting effect on the alcohol consumption progression scale in a way that it could influence the student being in an unhealthier category (such as moderate drinker) and discourage the student from being in an even more unhealthy category (such as binge drinker). Across all estimations, only two examples of this pattern were observed. These cases occur in the model that combines both male and female students, relating to the context of a student being a female or living in the southern region of Brazil.

Of course, some exceptions are acknowledged. As pointed out before, our results indicate an apparently conflicting behavior in relation to the student’s sex. The model estimated with boys and girls combined suggests that female students present a lower chance of experimenting with alcoholic beverages but a higher chance of exhibiting unhealthier profiles of alcohol consumption, such as moderate or binge drinking.

This result may be related to behavioral traits and social stigma linked to womanhood. Nolen-Hoeksema [27] indicates that women are less likely to show personality traits linked to alcohol consumption, such aggressiveness and antisociality. Nolen-Hoeksema [27] also indicates that women are more prone to adverse consequences linked to alcohol consumption, like requiring less exposition to develop alcohol-induced diseases and greater decline of cognitive and motor skills.

Likewise, Li et al. [28] state that girls usually report feeling more monitored by parents than boys and that the perceived parental monitoring tends to decrease with age. McArdle et al. [57] and Guo et al. [58] point out that an affective bond between parents and children contributes to limiting alcohol consumption. The combination of this evidence may explain the conflicting result, considering that a negative effect was observed on the transition that reflects alcohol initiation, which may take place at younger ages, and that being a girl was associated with a higher chance of moderate and binge drinking, which may be more commonly observed in more advanced ages of adolescence.

Another important result related to a possible gender bias was observed on the effect of time dedicated to physical activity. The model estimated only with female students indicated that physical activities did not influence girls’ alcohol consumption whereas the boys’ model suggests that more time performing physical activities might increase the odds of unhealthy alcohol consumption. Kwan et al. [59] systematically reviewed longitudinal studies analyzing sport participation and alcohol and illicit drug use in adolescents, and found that practicing sports is positively associated with alcohol consumption. Hoffman [60] suggests that being involved in sports may influence alcohol consumption due to socialization and cultural behaviors connected to sport activities. Wichstrøm and Wichstrøm [61] point out the existence of differences according to the type of sport. According to Wichstrøm and Wichstrøm [61], while endurance and individual sports reduce consumption, technical and team sports have been shown to increase the proportion of substance use in adolescence. Unfortunately, PeNSE 2015 does not consider the type of physical activity a student performs. Considering the importance that this detail may play in influencing adolescent alcohol consumption in Brazil, further research should focus on investigating the influence of different modalities of physical activity on teenage alcohol consumption.

Furthermore, looking at the results that relate to both masculine and feminine students, an important result concerns the influence of characteristics that may facilitate a student’s access to alcoholic beverages, in particular, the household economic status and whether a student takes up a paid occupation.

Regarding household economic status, Martins-Oliveira et al. [62] and Hanson and Chen [63] indicate the absence of consensus in the literature. Luthar and Becker [64] and Luthar and Latendresse [65] indicate that adolescents whose families possess greater economic conditions may be at greater risk of participating in risky health behaviors due to a combination of greater availability of disposable income, higher achievement pressures, and less adult supervision.

Our results appear to be in sync with those of Luthar and Becker [64] and Luthar and Latendresse [65] since they show that living in a household with greater economic conditions was positively associated with unhealthier profiles of alcohol consumption. A possible explanation for that result might be linked to Kumar et al. [33] remark that the combination of greater economic conditions and a more permissive environment may be what encourages adolescents with higher economic conditions to drink alcoholic beverages. Ibitoye et al. [66] studied the concentration of outdoor alcohol advertising and liquor stores near places where teenagers get together and posit that the absence of underage drinking laws enforcement might encourage teenage alcohol consumption.

Another result that deserves attention is the variable of employment in a paid occupation, like a job or internship. Our results show that students in the paid employment category presented a higher chance of being in unhealthier alcohol consumption categories. According to Kuntsche et al. [67], once students have greater financial resources, complementary to the socioeconomic status analysis, and in an environment characterized by greater interaction with older persons, their alcohol consumption increases. The very fact of drinking also affects the labor market. A review of some studies relative to the adult labor market outcomes and alcohol outcomes suggests the absence of a consensus between wages, employment rates, and alcohol consumption. Dave and Kaestner [68] results on US adults suggest that alcohol consumption does not negatively affect labor market outcomes. Böckerman et al. [69] twin data longitudinal analysis of Finnish men and women indicates that former drinkers and heavy drinkers have approximately 20% lower earnings when compared to moderate drinkers. Teckin [70] longitudinal analysis of the Russian population indicates a small linear effect on wage rates. Further research focused on adolescent alcohol consumption and labor market outcomes might provide new important evidence regarding the relation between them. In this context, data from the 2019 PNS showed that alcohol consumption tends to change according to per capita income, where the higher the income, the higher the proportion of people who consume alcoholic beverages. The rate of people who consume alcoholic beverages is 18.6% among those with no income, 27.8% among those earning between 1 to 2 salaries, and 49% among those earning more than 5 salaries [18].

Lastly, we recommend some caution when interpreting the results concerning the variables related to the number of friends who also consume alcohol, the consumption of tobacco-based products and illicit drugs, and mental health conditions. Although the first two groups of variables presented elevated odds ratio, meaning that consumption of other drugs and peer effects were highly associated with unhealthier alcohol consumption categories, previous studies have already indicated that these characteristics might be correlated with other unobserved aspects of a student’s life.

Many studies have shown the existence of an association between consumption of alcohol, tobacco, and other drugs (examples include [71–73]). Kandel [74] and Kandel et al. [75] gateway drug theory suggest the existence of an ordered sequence of stages in which the consumption of soft drugs (alcohol and tobacco) leads to consumption of harder drugs. Comparing the results regarding the relation between alcohol and tobacco and alcohol and illicit drugs, the greater odd ratio associated with the consumption of tobacco-based products could be interpreted as a possible outcome of a gateway effect. Nonetheless, French and Popovici [76] indicate that the consumption of addictive substances may be linked to unobserved characteristics, like preferences and attitudes towards risk and drugs. In this regard, it is important to emphasize that our results also suggest that alcohol prevention strategies may present a positive side effect in reducing the consumption of other drugs.

Regarding peer effects, McCann et al. [77] suggest that adolescents tend to imitate their peers’ level of alcohol consumption. Ali et al. [32] indicate that this behavior may be related to social motivations such as wanting to be popular or to be accepted in a new group of friends. Nesi et al. [78] state that exposure to social media, where users often tend to compare their own lives to others, may predict adolescent alcohol consumption. In that regard, Fletcher [79] notes that it is difficult to distinguish the group effect on an individual from the effect an individual may have on a group. Fletcher [79] indicates that the simultaneity arising from trying to determine who influences who makes the analysis of peer effects a not-so-straightforward task. Considering that adolescent drinking is an ongoing problem, this result suggests that public policies aimed at adolescent alcohol consumption prevention should have better effects when focused on a group level.

## 6. Conclusions

In this paper, we analyzed how various socioeconomic determinants might influence the alcohol consumption profile of ninth grade students in Brazil. Our analysis draws upon the literature focused on describing adolescent alcohol consumption in Brazil to investigate the existence of possible differences related to adolescent drinking based on the number of doses an adolescent consumes on the occasions when he or she consumes alcohol. Our results show that for a major part, the characteristics associated with different drinking profiles remain in the same direction. An exception is found in the context of gender. We found that female students present a lesser chance of experimenting with alcohol, however, they also present a higher chance of falling into an unhealthier pattern of drinking. Based on these results, our study’s main contribution to the literature and to the formulation of public policies is the suggestion to adopt a “one size fits all” approach because the different groups of alcohol consumption studied presented a similar profile. The very knowledge that adolescents first experimenting with alcoholic beverages and binge drinking adolescents happen to have remarkably similar characteristics raises the possibility of simplifying public policies aimed at this problem.

For future research, we suggest two advancements. First, the analysis of longitudinal data composed of both adolescents enrolled and not enrolled in school. The analysis of such data might show important differences on the determinants of adolescent alcohol consumption, especially considering that being enrolled in the education system may be a protective factor in limiting adolescent alcohol initiation and binge drinking. Second, from an analytical point of view, a further integrated analysis combining more elements related to alcohol consumption could be carried out. This might include aspects of intensity (how much alcohol an individual takes with each dose) and other consumption habits (like how many times a month one drinks alcohol and in which environments an adolescent consumes alcohol) that may further contribute to the identification of unhealthier habits of alcohol consumption and add more layers of precision for the formulation of public policies aimed at preventing underage drinking (in all of its possible forms) and reducing the negative side effects of continued alcohol consumption and abuse.

## Supporting information

S1 Table Description of variables. Source: Prepared by the authors based on information from PeNSE 2015.
(DOCX)

S2 Table Description of the results of the principal component analysis. Source: Prepared by the authors based on information from PeNSE 2015.
(DOCX)

S3 Table Brant [56] test results. Source: Prepared by the authors based on information from PeNSE 2015.
(DOCX)

S4 Table Description of the sample by alcohol consumption categories. Source: Prepared by the authors based on information from PeNSE 2015.
(DOCX)
