# Supplementary material for: What factors matter in the amount of alcohol consumed? An analysis among Brazilian adolescents
Source: PLoS One. 2023 Feb 21;18(2):e0281065. doi: 10.1371/journal.pone.0281065 (PMC9942966; doi:10.1371/journal.pone.0281065)
Supplement: S1 Table — Source: Prepared by the authors based on information from PeNSE 2015. (DOCX) [file pone.0281065.s001.docx]

**APPENDIX**

**Table A.1** – Description of variables

| **Variable** | **Description** |
| --- | --- |
| Sex (girls) | Equal to one if the student declared to be female. Zero if otherwise. |
| Economic condition | Summary variable to describe the economic condition of the student and his/her home. Formulated from the analysis of the main components of the variables (assumes continuous values of the main component):   - Landline telephone: equal to one if the student's home has a landline telephone (zero if otherwise). - Mobile phone: equal to one if the student has a mobile phone for individual use (zero if otherwise). - Computer: equal to one if the student's home has a computer of any kind (desktop, laptop, etc.) (zero if otherwise). - Internet access: equal to one if the student has internet access at home (zero if otherwise). - Car: equal to one if any resident of the student's home has a car (zero if otherwise). - Motorcycle: equal to one if a person from the student's household owns a motorcycle (zero if otherwise). - Housekeeper: equal to one if the student's home has a housekeeper receiving money to do domestic activities three or more days a week (zero if otherwise). |
| Student’s age | Students’ age categories |
| Age less than or equal to 13 years | Equal to one if the student is 13 years old or less.  Category omitted from the estimation. |
| Age between 14 and 17 years | Equal to one if the student is between 14 and 17 years old. Zero if otherwise. |
| Age 18 years or older | Equal to one if the student is 18 years or older. Zero if otherwise. |
| Racial group | Student ethnicity categories |
| Caucasian | Equal to one if the student declared to be Caucasian. Category omitted from the estimation. |
| Black | Equal to one if the student declared to be Black. Zero if otherwise. |
| Asian | Equal to one if the student declared to be Asian. Zero if otherwise. |
| Multiracial | Equal to one if the student declared to be Multiracial. Zero if otherwise. |
| Indigenous (native) | Equal to one if the student declared to be Indigenous (native). Zero if otherwise. |
| Activities and occupations | Categories indicating the participation or not of students in extracurricular occupations related or not to the labor market |
| Only studies | Equal to one if the student does not have his or her own business, work, or other occupation besides studying. Category omitted from the estimation. |
| Studies and takes up unpaid occupation | The same as if the student declared having his or her own business or another occupation besides studying but does not receive remuneration (money) for doing this activity. Zero if otherwise. |
| Studies and takes up paid occupation | The same as if the student declared having his or her own business or another occupation besides studying and receives remuneration (money) for doing this activity. Zero if otherwise. |
| Time of extracurricular physical activity | Time (in hours) spent on physical activities (playing sports, dancing, exercising at a gym, etc.) outside the school's Physical Education (PE) classes. Zero if the student does not perform any type of extracurricular physical activity. |
| Consumption of tobacco-based products | Equal to one if the student declared that he or she had consumed cigarettes or other tobacco-based products in the thirty days prior to the application of the survey. Zero if otherwise. |
| Consumption of illicit drugs | Equal to one if the student declared having consumed illicit drugs (marijuana, LSD, crack, etc.) in the thirty days prior to the application of the research. Zero if otherwise. |
| Emotional state | - Summary variable to portray the level of mental or psychological well-being of the student. Formulated from the analysis of the main components of the variables (assumes continuous values of the main component): - Frequency at which the student felt upset, bothered, hurt, offended, or humiliated by schoolmates: continuous scale of frequency of times when the student felt upset, bothered, hurt, offended, or humiliated by schoolmates in the thirty days prior to the application of the survey. (1 = never, 2 = rarely, 3 = sometimes, 4 = most of the time, 5 = always.) - Frequency at which the student felt alone: continuous scale of frequency of times when he or she felt alone in the twelve months prior to the application of the survey. (1 = never, 2 = rarely, 3 = sometimes, 4 = most of the time, 5 = always.) - Frequency in which the student was unable to sleep at night because something concerned him or her a lot: continuous scale of frequency of times when the student was unable to sleep at night because something worried him or her in the twelve months prior to the application of the survey. (1 = never, 2 = rarely, 3 = sometimes, 4 = most of the time, 5 = always.) |
| Number of friends who consume alcoholic beverages | Categories indicating the number of school friends who consume alcoholic beverages. |
| None of the friends consume alcoholic beverages | Equal to one if the student declared that none of the friends consumes alcoholic beverages. Category omitted from estimation. |
| Few friends consume alcoholic beverages | Equal to one if the student declared that few friends consume alcoholic beverages. Zero if otherwise. |
| Some friends consume alcoholic beverages | Equal to one if the student declared that some of his/her friends consume alcoholic beverages. Zero if otherwise. |
| Majority of friends consume alcoholic beverages | Equal to one if the student declared that most of his/her friends consume alcoholic beverages. Zero if otherwise. |
| All friends consume alcoholic beverages | Equal to one if the student declared that all friends consume alcoholic beverages. Zero if otherwise. |
| Public school | Equal to one if the student studies in a public school. Zero if otherwise. |
| Full time | Equal to one if the student studies full time (has school activities for seven or more hours a day throughout the school period). Zero otherwise. |
| Family composition | Categories indicating the family composition of the students. |
| Does not live with any parent or guardian | Equal to one if the student does not live in the same household as the parent or guardian. Category omitted from the estimation. |
| Lives only with one parent or guardian | Equal to one if the student lives in the same household as one of the parents or guardians. Zero if otherwise. |
| Lives with both parents or guardians | Equal to one if the student lives in the same household as both parents or guardians. Zero if otherwise. |
| Level of supervision of parents or guardians | - Summary variable to portray the level of supervision or attention paid by parents or guardians to the student's behavior. Formulated from the analysis of the main components of the variables (assumes continuous values of the main component): - Frequency at which parents or guardians knew what the student was doing in free time: continuous scale of frequency of times that parents or guardians knew what the student was doing in free time in the thirty days prior to the application of the survey. (1 = never, 2 = rarely, 3 = sometimes, 4 = most of the time, 5 = always.) - Frequency at which the parents or guardians checked the student’s homework: continuous scale of frequency of times the parents or guardians checked the student’s homework in the thirty days prior to the application of the survey. (1 = never, 2 = rarely, 3 = sometimes, 4 = most of the time, 5 = always.) - Frequency at which the parents or guardians went through the student's things without their permission: continuous scale of frequency of times that parents or guardians went through the student’s belongings without their permission in the thirty days prior to the application of the survey. (1 = never, 2 = rarely, 3 = sometimes, 4 = most of the time, 5 = always.) |
| Number of residents in the household | Number of people (excluding the student) living in the same household as the student. Assumes a value of zero if the student lives alone. |
| Geographic region | Categories indicating the geographic location of the school among the five administrative regions that internally divide the Brazilian territory. |
| North | Equal to one if the school is located in the North of Brazil (region comprised of the states of Acre, Amapá, Amazonas, Pará, Rondônia, Roraima, and Tocantins). Category omitted from the estimation. |
| Northeast | Equal to one if the school is located in the Northeast region of Brazil (region composed of the states of Alagoas, Bahia, Ceará, Maranhão, Paraíba, Piauí, Pernambuco, Rio Grande do Norte, and Sergipe). Zero if otherwise. |
| Southeast | Equal to one if the school is located in the Southeast region of Brazil (region composed of the states of Espírito Santo, Minas Gerais, Rio de Janeiro, and São Paulo). Zero if otherwise. |
| South | Equal to one if the school is located in the South region of Brazil (a region composed of the states of Rio Grande do Sul, Santa Catarina, and Paraná). Zero if otherwise. |
| Midwest | Equal to one if the school is located in the Midwest region of Brazil (a region composed of the Federal District and the states of Goiás, Mato Grosso, and Mato Grosso do Sul). Zero if otherwise. |

Source: Prepared by the authors based on information from PeNSE 2015
