# Supplementary material for: What factors matter in the amount of alcohol consumed? An analysis among Brazilian adolescents
Source: PLoS One. 2023 Feb 21;18(2):e0281065. doi: 10.1371/journal.pone.0281065 (PMC9942966; doi:10.1371/journal.pone.0281065)
Supplement: S2 Table — Source: Prepared by the authors based on information from PeNSE 2015. (DOCX) [file pone.0281065.s002.docx]

**APPENDIX**

**Table A.2** – Description of the results of the principal component analysis

| PCA – Economic condition | | |  |  |
| --- | --- | --- | --- | --- |
| Component | Eigenvalue | Difference | Proportion | Cumulative |
| Comp1 | 222002 | 120244 | 0.3171 | 0.3171 |
| Comp2 | 101758 | 0.0278704 | 0.1454 | 0.4625 |
| Comp3 | 0.98971 | 0.108604 | 0.1414 | 0.6039 |
| Comp4 | 0.881106 | 0.15117 | 0.1259 | 0.7298 |
| Comp5 | 0.729936 | 0.0490523 | 0.1043 | 0.8341 |
| Comp6 | 0.680884 | 0.200118 | 0.0973 | 0.9313 |
| Comp7 | 0.480766 | 0 | 0.0687 | 1 |
|  |  |  |  |  |
| PCA – Parents’ supervision | |  |  |  |
| Component | Eigenvalue | Difference | Proportion | Cumulative |
| Comp1 | 126.547 | 0.265288 | 0.4218 | 0.4218 |
| Comp2 | 100.018 | 0.265829 | 0.3334 | 0.7552 |
| Comp3 | 0.734352 | 0 | 0.2448 | 1 |
|  |  |  |  |  |
| PCA – Emotional state | |  |  |  |
| Component | Eigenvalue | Difference | Proportion | Cumulative |
| Comp1 | 160.704 | 0.74028 | 0.5357 | 0.5357 |
| Comp2 | 0.866761 | 0.340564 | 0.2889 | 0.8246 |
| Comp3 | 0.526197 | 0 | 0.1754 | 1 |

Source: Prepared by the authors based on the estimation of the results
