# Supplementary material for: What factors matter in the amount of alcohol consumed? An analysis among Brazilian adolescents
Source: PLoS One. 2023 Feb 21;18(2):e0281065. doi: 10.1371/journal.pone.0281065 (PMC9942966; doi:10.1371/journal.pone.0281065)
Supplement: S3 Table — Source: Prepared by the authors based on information from PeNSE 2015. (DOCX) [file pone.0281065.s003.docx]

**APPENDIX**

**Table A.3** – Brant [56] test results

|  | General | | Female (girls) | | Male (boys) | |
| --- | --- | --- | --- | --- | --- | --- |
| Statistic | Test Statistic (chi-squared) | p value | Test Statistic (chi-squared) | p value | Test Statistic (chi-squared) | p value |
|  |  |  |  |  |  |  |
| Model result | 1518.80 | 0.000 | 794.52 | 0.000 | 728.79 | 0.000 |
|  |  |  |  |  |  |  |
| Variables Result |  |  |  |  |  |  |
|  |  |  |  |  |  |  |
| Sex (girls) | 144.14 | 0.000 | (-) | (-) | (-) | (-) |
| Economic condition | 30.82 | 0.000 | 12.67 | 0.002 | 19.23 | 0.000 |
| Student’s age |  |  |  |  |  |  |
| Age less than or equal to 13 years (base category) | (-) | (-) | (-) | (-) | (-) | (-) |
| Age between 14 and 17 years | 18.86 | 0.000 | 6.80 | 0.033 | 17.95 | 0.000 |
| Age 18 years or older | 2.23 | 0.143 | 6.93 | 0.039 | 2.99 | 0.225 |
| Racial group |  |  |  |  |  |  |
| Caucasian (base category) | (-) | (-) | (-) | (-) | (-) | (-) |
| Black | 4.82 | 0.090 | 0.93 | 0.628 | 10.90 | 0.004 |
| Asian | 9.32 | 0.009 | 5.09 | 0.079 | 4.67 | 0.097 |
| Multiracial | 6.51 | 0.039 | 0.16 | 0.925 | 13.92 | 0.001 |
| Indigenous (native) | 3.25 | 0.197 | 1.31 | 0.519 | 3.52 | 0.172 |
| Activities or occupations |  |  |  |  |  |  |
| Only studies (base category) | (-) | (-) | (-) | (-) | (-) | (-) |
| Studies and takes up unpaid occupation | 2.22 | 0.330 | 0.68 | 0.711 | 3.75 | 0.153 |
| Studies and takes up paid occupation | 0.03 | 0.985 | 6.98 | 0.030 | 3.75 | 0.154 |
| Time of extracurricular physical activity | 17.84 | 0.000 | 16.55 | 0.000 | 16.30 | 0.000 |
| Consumption of tobacco-based products | 78.88 | 0.000 | 62.03 | 0.000 | 32.86 | 0.000 |
| Consumption of illicit drugs | 77.50 | 0.000 | 29.73 | 0.000 | 48.73 | 0.000 |
| Emotional state | 81.96 | 0.000 | 47.07 | 0.000 | 41.13 | 0.000 |
| Number of friends who consume alcoholic beverages |  |  |  |  |  |  |
| None of the friends consume alcoholic beverages (basic category) | (-) | (-) | (-) | (-) | (-) | (-) |
| Few friends consume alcoholic beverages | 46.24 | 0.000 | 28.23 | 0.000 | 20.36 | 0.000 |
| Some friends consume alcoholic beverages | 77.18 | 0.000 | 36.33 | 0.000 | 44.16 | 0.000 |
| Majority of friends consume alcoholic beverages | 145.32 | 0.000 | 70.24 | 0.000 | 77.63 | 0.000 |
| All friends consume alcoholic beverages | 97.60 | 0.000 | 43.93 | 0.000 | 56.56 | 0.000 |
| Public school | 7.04 | 0.030 | 3.31 | 0.191 | 5.13 | 0.077 |
| Full time | 1.86 | 0.396 | 6.92 | 0.031 | 2.53 | 0.282 |
| Family composition |  |  |  |  |  |  |
| Does not live with either parent or guardian (base category) | (-) | (-) | (-) | (-) | (-) | (-) |
| Lives with only one parent or guardian | 4.64 | 0.098 | 4.13 | 0.127 | 1.08 | 0.583 |
| Lives with both parents or guardians | 46.07 | 0.000 | 29.94 | 0.000 | 13.94 | 0.001 |
| Level of supervision of parents or guardians | 238.53 | 0.000 | 135.80 | 0.000 | 103.53 | 0.000 |
| Number of residents in the household | 6.02 | 0.049 | 5.49 | 0.064 | 2.20 | 0.332 |
| Geographic region |  |  |  |  |  |  |
| North (base category) | (-) | (-) | (-) | (-) | (-) | (-) |
| Northeast | 66.49 | 0.000 | 38.03 | 0.000 | 33.42 | 0.000 |
| Southeast | 18.69 | 0.000 | 14.05 | 0.001 | 8.24 | 0.016 |
| South | 40.07 | 0.000 | 31.33 | 0.000 | 11.45 | 0.003 |
| Midwest | 6.34 | 0.042 | 3.37 | 0.185 | 3.73 | 0.155 |

Source: Prepared by the authors based on the test results

Note: $H_{0}$: the proportionality of chances is equal to each stage. (-) denotes variable not included in the model.
