# Supplementary material for: What factors matter in the amount of alcohol consumed? An analysis among Brazilian adolescents
Source: PLoS One. 2023 Feb 21;18(2):e0281065. doi: 10.1371/journal.pone.0281065 (PMC9942966; doi:10.1371/journal.pone.0281065)
Supplement: S4 Table — Source: Prepared by the authors based on information from PeNSE 2015. (DOCX) [file pone.0281065.s004.docx]

**APPENDIX**

**Table A.4** – Description of the sample by alcohol consumption categories

|  | Never-Drinkers | Nondrinkers | Moderate Drinkers | Binge Drinkers | Total |
| --- | --- | --- | --- | --- | --- |
| Sex |  |  |  |  |  |
| Boys | 22,522 | 14,719 | 8,738 | 2,305 | 48,284 |
|  | 46.6% | 30.5% | 18.1% | 4.8% |  |
| Girls | 23,556 | 14,869 | 10,239 | 3,277 | 51,941 |
|  | 45.4% | 28.6% | 19.7% | 6.3% |  |
| Household economic status |  |  |  |  |  |
| Mean | -0.053 | 0.011 | 0.045 | 0.232 |  |
| Std Dev | 1.525 | 1.458 | 1.472 | 1.394 |  |
| Age groups |  |  |  |  |  |
| 13 years old or less | 9,793 | 4,455 | 2,259 | 410 | 16,917 |
|  | 57.9% | 26.3% | 13.4% | 2.4% |  |
| Between 14 and 17 years old | 35,886 | 24,714 | 16,368 | 5,050 | 82,018 |
|  | 43.8% | 30.1% | 20.0% | 6.2% |  |
| 18 years old or more | 399 | 419 | 350 | 122 | 1,290 |
|  | 30.9% | 32.5% | 27.1% | 9.5% |  |
| Racial group |  |  |  |  |  |
| Caucasian | 15,509 | 9,568 | 6,364 | 1,819 | 33,260 |
|  | 46.6% | 28.8% | 19.1% | 5.5% |  |
| Black | 5,400 | 3,767 | 2,539 | 826 | 12,532 |
|  | 43.1% | 30.1% | 20.3% | 6.6% |  |
| Asian | 1,992 | 1,407 | 821 | 275 | 4,495 |
|  | 44.3% | 31.3% | 18.3% | 6.1% |  |
| Multiracial | 21,430 | 13,697 | 8,539 | 2,445 | 46,111 |
|  | 46.5% | 29.7% | 18.5% | 5.3% |  |
| Indigenous | 1,691 | 1,121 | 699 | 214 | 3,725 |
|  | 45.4% | 30.1% | 18.8% | 5.7% |  |
| Extracurricular activities |  |  |  |  |  |
| Only Studies | 42,263 | 25,630 | 15,518 | 4,234 | 87,645 |
|  | 48.2% | 29.2% | 17.7% | 4.8% |  |
| Studies and unpaid occupation | 382 | 344 | 256 | 72 | 1,054 |
|  | 36.2% | 32.6% | 24.3% | 6.8% |  |
| Studies and paid occupation | 3,411 | 3,597 | 3,195 | 1,273 | 11,476 |
|  | 29.7% | 31.3% | 27.8% | 11.1% |  |
| Time spent on physical activities |  |  |  |  |  |
| Mean | 1.870 | 2.018 | 2.096 | 2.341 |  |
| Std Dev | 2.369 | 2.500 | 2.517 | 2.717 |  |
| Tobacco-based product consumption |  |  |  |  |  |
| No | 45,654 | 28,147 | 15,260 | 3,268 | 92,329 |
|  | 49.4% | 30.5% | 16.5% | 3.5% |  |
| Yes | 415 | 1,432 | 3,713 | 2,308 | 7,868 |
|  | 5.3% | 18.2% | 47.2% | 29.3% |  |
| Illicit drug use |  |  |  |  |  |
| No | 45,932 | 28,989 | 17,193 | 4,164 | 96,278 |
|  | 47.7% | 30.1% | 17.9% | 4.3% |  |
| Yes | 106 | 557 | 1,751 | 1,398 | 3,812 |
|  | 2.8% | 14.6% | 45.9% | 36.7% |  |
| Number of friends who drink alcoholic beverages |  |  |  |  |  |
| None | 15,421 | 3,833 | 843 | 84 | 20,181 |
|  | 76.4% | 19.0% | 4.2% | 0.4% |  |
| A few | 13,301 | 9,401 | 4,563 | 525 | 27,790 |
|  | 47.9% | 33.8% | 16.4% | 1.9% |  |
| Some | 8,387 | 8,487 | 5,705 | 1,139 | 23,718 |
|  | 35.4% | 35.8% | 24.1% | 4.8% |  |
| Most | 3,167 | 4,991 | 6,024 | 2,849 | 17,031 |
|  | 19% | 29% | 35% | 17% |  |
| All | 317 | 575 | 1,025 | 820 | 2,737 |
|  | 11.6% | 21.0% | 37.4% | 30.0% |  |
| Mental health condition |  |  |  |  |  |
| Mean | -0.247 | 0.109 | 0.283 | 0.497 |  |
| Std Dev | 1.209 | 1.256 | 1.270 | 1.365 |  |
| School management |  |  |  |  |  |
| Private School | 10,237 | 5,904 | 3,516 | 1,095 | 20,752 |
|  | 49.3% | 28.5% | 16.9% | 5.3% |  |
| Public School | 35,841 | 23,684 | 15,461 | 4,487 | 79,473 |
|  | 45.1% | 29.8% | 19.5% | 5.6% |  |
| Full time school |  |  |  |  |  |
| No | 35,586 | 23,157 | 14,508 | 4,298 | 77,549 |
|  | 45.8% | 29.8% | 18.7% | 5.5% |  |
| Yes | 10,232 | 6,312 | 4413 | 1,264 | 22,221 |
|  | 46.0% | 28.4% | 19.8% | 5.6% |  |
| Household family structure |  |  |  |  |  |
| Does not live in the same household | 2,276 | 2,192 | 1,452 | 551 | 6,471 |
|  | 35.2% | 33.9% | 22.4% | 8.5% |  |
| Lives with one parent (mother or father) | 14,337 | 11,632 | 7,526 | 2,472 | 35,967 |
|  | 39.9% | 32.3% | 20.9% | 6.9% |  |
| Lives with both parents | 29,404 | 15,737 | 9978 | 2,555 | 57,674 |
|  | 51.0% | 27.3% | 17.3% | 4.4% |  |
| Parental supervision |  |  |  |  |  |
| Mean | 0.246 | -0.148 | -0.238 | -0.438 |  |
| Std Dev | 1.087 | 1.104 | 1.113 | 1.138 |  |
| Number of people living in the same household |  |  |  |  |  |
| Mean | 4.588 | 4.497 | 4.513 | 4.413 |  |
| Std Dev | 2.045 | 1.936 | 2.250 | 1.681 |  |
| Geographic region |  |  |  |  |  |
| North | 11,171 | 6,985 | 3,814 | 1,034 | 23,004 |
|  | 48.6% | 30.4% | 16.6% | 4.5% |  |
| Northeast | 17,938 | 10,011 | 6,217 | 1,791 | 35,957 |
|  | 49.9% | 27.8% | 17.3% | 5.0% |  |
| Southeast | 7,820 | 5,132 | 3,455 | 1,094 | 17,501 |
|  | 44.7% | 29.3% | 19.7% | 6.3% |  |
| South | 3,384 | 3,026 | 2,596 | 741 | 9,747 |
|  | 34.7% | 31.0% | 26.6% | 7.6% |  |
| Midwest | 5,765 | 4,434 | 2895 | 922 | 14,016 |
|  | 41.1% | 31.6% | 20.7% | 6.6% |  |
| Total | 46,079 | 29,588 | 18,977 | 5,582 | 100,225 |
|  | 45.9% | 29.5% | 18.9% | 5.5% |  |

Source: Prepared by the authors based on information from PeNSE 2015
